# American Edition of Nothnagel’s Practice

**Published:** 1903-02

**Authors:** 


American Edition of Nothnagel's Practice.—Diseases of the Bronchi and Pleura; Pneumonia. Diseases of the Bronchi, by Dr. F. A. Hoffmann, of Leipsic. Diseases of the Pleura, by Dr. O. Rosenbach, of Berlin. Pneumonia, by Dr. F. Aufrecht, of Magdeburg. Edited, with editions, by John H. Musser, M. D., Professor of Clinical Medicine, University of Pennsylvania. Handsome octavo volume of 1030 pages, illustrated, including 7 full-page colored lithographic plates. Philadelphia and London: W. B. Saunders & Co., 1902. Cloth, $5.00, net; half morocco, $6.00, net.

This volume stands out pre-eminently the leader of its class, embracing, as it does, all the knowledge of today of diseases of the bronchi, lungs and pleura, described in a most interesting and comprehensive manner, with a simple classification and easy reference. This book includes not only the valuable monographs of its eminent authors, which alone, with their breadth of learning, their exhaustive research and extensive practical experience, would be ample recommendation, but contains new matter on anatomy and physiology of the bronchi, also pathology, bacteriology and treatment of bronchitis, and the recent researches on bronchiectasis and on eosinophilia in asthma.

The first 219 pages are devoted to the bronchi, including anatomy, physiology, malformations, injuries, foreign bodies, forms of bronchitis, tumors, bronchiectasis. traction-diverticular, perforation and asthma, while under each heading is included morbid anatomy, etiology, course, symptomology, complications, prognosis, treatment and diagnosis. Emphysema and atelectasis comprise the next 175 pages. The clinical picture of the emphysema is cloaked in easily understood language, and brings out some new ideas relating to the shape of the emphysematous chest in children.

The lungs are considered in the next 410 pages, in which inflammatory conditions extensively are discussed. Tuberculosis is to be considered in another volume. This section in dealing with pneumonia incorporates the recent work of Hutchinson and others on the blood and urine, which are most valuable contributions, as well as the author's own conclusions. The chapter on treatment interestingly discusses all methods in vogue by various eminent authorities, but fails to reduce the mortality from its high place of about 25 per cent., which it has so vigorously maintained for the past century.

As regards serum-therapy, the author says, "it remains for later investigators to find out if it will be possible, with the help of the antitoxins of the pneumococcus, to cure pneumonia and so make unnecessary all methods of treatment in use at the present time." He, however, gives space to the most important attempts in that direction.

The last section contains 318 pages, devoted to diseases of the pleura. The chapter on bacterial etiology is especially instructive, giving a complete resume of the work done by various noted authors. The blood changes as indicated by Morse, or rather the absence of leucocytosis, is of value in differential diagnosis. "If a leucocytosis exists with pleurisy there is probably a complicating pneumonia." In the early treatment of pleurisy the salicylates in large doses are strongly recommended. The same style of easy and forceful expression is continued throughout the volume, and throughout the entire book are numerous quotations from American authors.

The plates that this book contains cannot be improved upon and the other illustrations are excellent.

There are a number of valuable tables.

The intrinsic worth of this volume can only be comprehended by a careful reading and digesting of its contents, every word of which is worth reading. Time and money are well spent when such a fruitful store of knowledge can be so easily obtained.

